# *rac*-{[2-(Diphenyl­thio­phosphan­yl)ferrocen­yl]meth­yl}trimethyl­ammonium iodide chloro­form monosolvate

**DOI:** 10.1107/S1600536812046053

**Published:** 2012-11-17

**Authors:** Andrei Karpous, Zoia Voitenko, Jean-Claude Daran, Eric Manoury

**Affiliations:** a CNRS, LCC, 205 route de Narbonne, BP 44099, F-31077, Toulouse Cedex 4, France; b University Taras Shevchenko, Vladimirska st 64, 01033 Kiev, Ukraine

## Abstract

The title compound, [Fe(C_5_H_5_)(C_21_H_24_NPS)]I·CHCl_3_, is built up from a (ferrocenylmeth­yl)trimethyl­ammonium cation, a iodine anion and a chloro­form solvent mol­ecule, all residing in general positions. The N atom of the ammonium group is displaced by 1.182 (2) Å from the plane of the substituted cyclo­penta­dienyl (Cp) ring towards the Fe atom, whereas the C atom attached to the same Cp ring is slightly below this plane by −0.128 (2) Å. These deviations might result from weak agostic interactions between the two H atoms of the CH_2_ group and the Fe atom.

## Related literature

For related structures containing the (ferrocen­yl)trimethyl­ammonium framework, see: Bai *et al.* (2011 ▶); Ballester *et al.* (2003 ▶); Blake *et al.* (2004 ▶); Broomsgrove *et al.* (2010 ▶); Chohan *et al.* (1997 ▶); Deck *et al.* (2000 ▶); Ferguson *et al.* (1994 ▶); Herbstein & Kapon (2008 ▶); Hong *et al.* (2005 ▶); Hosmane *et al.* (1998 ▶); Hu *et al.* (2004 ▶); Li *et al.* (2009 ▶); Malezieux *et al.* (1994 ▶); Pullen *et al.* (1998 ▶); Reynes *et al.* (2002 ▶); Selvapalam *et al.* (2007 ▶); Sharma *et al.* (2006 ▶); Veya & Kochi (1995 ▶); Volkov *et al.* (2003 ▶, 2005 ▶, 2006 ▶); Xu *et al.* (2010 ▶); Yongmao *et al.* (1982 ▶); Zhuji *et al.* (1982 ▶). For their use in chemistry, see: Routaboul *et al.* (2005 ▶, 2007 ▶); Mateus *et al.* (2006 ▶); Le Roux *et al.* (2007 ▶); Diab *et al.* (2008 ▶); Audin *et al.* (2010 ▶); Debono *et al.* (2010 ▶). For a description of the Cambridge Structural Database, see: Allen (2002 ▶).
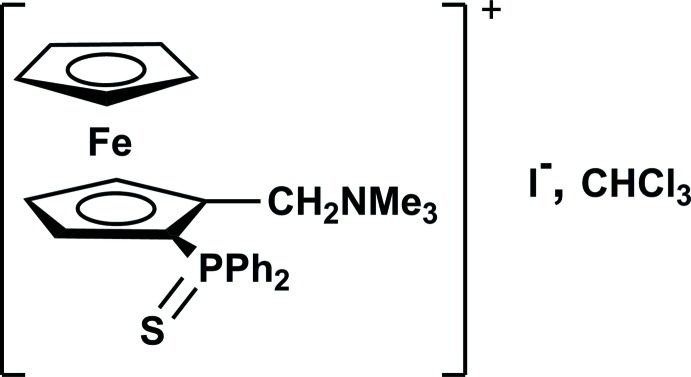


## Experimental

### 

#### Crystal data

[Fe(C_5_H_5_)(C_21_H_24_NPS)]I·CHCl_3_
*M* *_r_* = 720.65
Monoclinic, 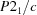

*a* = 17.4056 (6) Å
*b* = 12.1843 (3) Å
*c* = 14.9389 (5) Å
β = 110.632 (4)°
*V* = 2964.97 (18) Å^3^
*Z* = 4
Mo *K*α radiation
μ = 1.96 mm^−1^
*T* = 180 K
0.49 × 0.18 × 0.10 mm

#### Data collection

Agilent Xcalibur (Sapphire1, long nozzle) diffractometer
Absorption correction: multi-scan (*CrysAlis PRO*; Agilent, 2012 ▶) *T*_min_ = 0.574, *T*_max_ = 1.0
31103 measured reflections
6065 independent reflections
5385 reflections with *I* > 2σ(*I*)
*R*_int_ = 0.034

#### Refinement

*R*[*F* ^2^ > 2σ(*F* ^2^)] = 0.025
*wR*(*F* ^2^) = 0.062
*S* = 1.08
6065 reflections
319 parameters
H-atom parameters constrained
Δρ_max_ = 0.62 e Å^−3^
Δρ_min_ = −0.61 e Å^−3^

### 

Data collection: *CrysAlis PRO* (Agilent, 2012 ▶); cell refinement: *CrysAlis PRO*; data reduction: *CrysAlis PRO*; program(s) used to solve structure: *SIR97* (Altomare *et al.*, 1999 ▶); program(s) used to refine structure: *SHELXL97* (Sheldrick, 2008 ▶); molecular graphics: *ORTEPIII* (Burnett & Johnson, 1996 ▶) and *ORTEP-3 for Windows* (Farrugia, 2012 ▶); software used to prepare material for publication: *WinGX* (Farrugia, 2012 ▶).

## Supplementary Material

Crystal structure: contains datablock(s) I, global. DOI: 10.1107/S1600536812046053/rn2109sup1.cif

Structure factors: contains datablock(s) I. DOI: 10.1107/S1600536812046053/rn2109Isup2.hkl

Additional supplementary materials:  crystallographic information; 3D view; checkCIF report

## Figures and Tables

**Table 1:** Hydrogen-bond geometry (Å, °)

*D*—H⋯*A*	*D*—H	H⋯*A*	*D*⋯*A*	*D*—H⋯*A*
C24—H24*C*⋯I1	0.98	3.05	4.001 (3)	163
C100—H100⋯I1	1.00	2.93	3.810 (3)	147

**Fig. 1.**
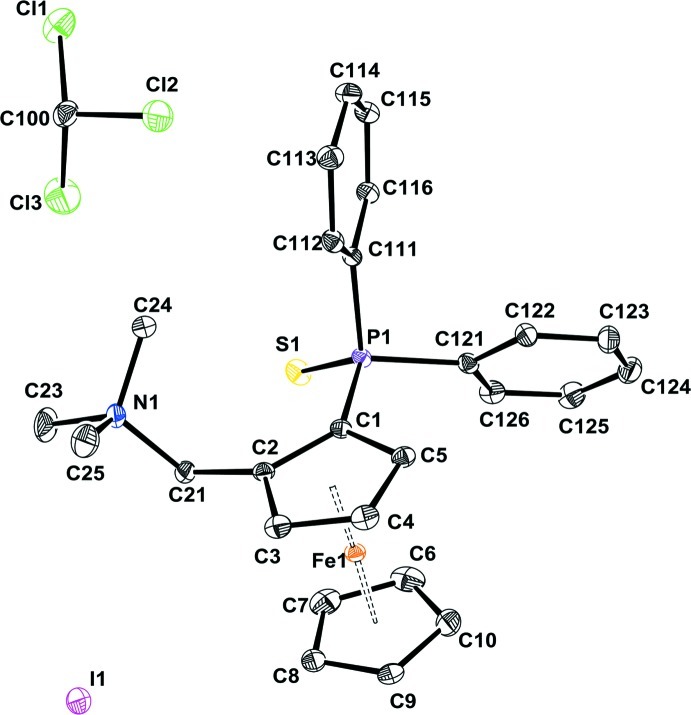
Molecular view of compound I with the atom labeling scheme. Ellipsoids are drawn at the 30% probability level. H atoms have been omitted for clarity.

## supplementary crystallographic information

### Comment

Recently, our group has synthesized various chiral enantiomerically pure ferrocenyl ligands and tested them in different catalytic asymmetric reactions (Routaboul *et al.*, 2005; Mateus *et al.*, 2006; Routaboul *et al.*, 2007; Le Roux *et al.*, 2007; Diab *et al.*, 2008; Audin *et al.*, 2010; Debono *et al.*, 2010). These ligands are synthesized from enantiomerically pure 2-(diphenylthiophosphanyl)(hydroxymethyl)ferrocene. One intermediate in the synthesis of such enantiomerically pure building block is the racemic (2-diphenylthiophosphanylferrocenyl) trimethylammonium iodide (Mateus *et al.*, 2006).

The asymmetric unit is built up from the (ferrocenylmethyl)trimethylammonium cation, the iodine anion and a chloroform molecule as solvate (Fig. 1). Except for the occurrence of the chloroform solvate, the structure is closely related to the one reported by Ferguson *et al.* (1994). However, in their case, the iodine was in weak interaction with one of the H atom of the bridging CH_2_ group whereas in our case the shortest interactions with the iodine involved one of the methyl of the ammonium and the H atom of the chloroform (Table 1). The phosphorus, P1 atom, is roughly in the plane of the Cp ring to which it is attached deviating only by -0.013 (1) Å whereas the sulfur, S1, is *endo* located -0.887 (1) Å below the Cp ring.

In the Cambridge Structural Database (CSD version 5.33, 2011; Allen, 2002), there are, to the best of our knowledge, 34 hits corresponding to structures involving the (ferrocenylmethyl)trimethylammonium cation with different counter ions. A comparison of selected distances and angles within the Cp—C-NMe_3_ framework is reported in supplementary materials. Surprisingly, there is no real influence of the counter ion on the geometry of this framework. In all compounds the bridging C *sp*^3^ atom is always *endo* with respect to the Cp ring to which it is attached with values ranging from -0.07 to -0.426 Å, whereas the ammonium N atom is always *exo* with values ranging from 0.999 to 1.914 Å. Surprisingly, these two extreme values are related to compound containing a very large anion, the (µ_12_-phosphato)-tetracosakis(µ_2_-oxo)-dodecaoxo-molybdenum(v)-undeca-molybdenum(vi) (Li *et al.*, 2009). However, it is worthwhile to note that the asymmetric unit in this polyoxomolybdate anions contains four molecules of which two of them have distance of the N from the Cp ring within the usual range: 1.248 and 1.152 Å. Moreover there are other compounds containing polyoxomolybdate anions (Xu *et al.*, 2010; Li *et al.* 2009) for which the values are within the normal range. So, these two extreme values might be the consequence of crystal packing which should accommodate four molecules within the asymetric unit.

### Experimental

(2-diphenylthiophosphanylferrocenyl) trimethylammonium iodide was synthesized by a published procedure (Mateus *et al.*, 2006). Single crystals suitable for X-ray diffraction analysis were grown from a chloroform solution by slow evaporation of the solvent.

### Refinement

All H atoms were fixed geometrically and treated as riding with C—H = 0.95 Å (aromatic), 0.98 Å (methyl), 0.99 Å (methylene) and 1.0 Å (methine) with *U*_iso_(H) = 1.2*U*_eq_(C) or *U*_iso_(H) = 1.5*U*_eq_(C_methyl_).

### Crystal data

**Table:**

[Fe(C_5_H_5_)(C_21_H_24_NPS)]I·CHCl_3_	*F*(000) = 1440
*M**_r_* = 720.65	*D*_x_ = 1.614 Mg m^−^^3^
Monoclinic, *P*2_1_/*c*	Mo *K*α radiation, λ = 0.71073 Å
Hall symbol: -P 2ybc	Cell parameters from 19830 reflections
*a* = 17.4056 (6) Å	θ = 2.9–28.4°
*b* = 12.1843 (3) Å	µ = 1.96 mm^−^^1^
*c* = 14.9389 (5) Å	*T* = 180 K
β = 110.632 (4)°	Box, yellow
*V* = 2964.97 (18) Å^3^	0.49 × 0.18 × 0.10 mm
*Z* = 4	

### Data collection

**Table:**

Agilent Xcalibur (Sapphire1, long nozzle) diffractometer	6065 independent reflections
Radiation source: fine-focus sealed tube	5385 reflections with *I* > 2σ(*I*)
Graphite monochromator	*R*_int_ = 0.034
Detector resolution: 8.2632 pixels mm^-1^	θ_max_ = 26.4°, θ_min_ = 2.9°
ω scans	*h* = −21→21
Absorption correction: multi-scan (*CrysAlis PRO*; Agilent, 2012)	*k* = −15→15
*T*_min_ = 0.574, *T*_max_ = 1.0	*l* = −18→18
31103 measured reflections	

### Refinement

**Table:**

Refinement on *F*^2^	Primary atom site location: structure-invariant direct methods
Least-squares matrix: full	Secondary atom site location: difference Fourier map
*R*[*F*^2^ > 2σ(*F*^2^)] = 0.025	Hydrogen site location: inferred from neighbouring sites
*wR*(*F*^2^) = 0.062	H-atom parameters constrained
*S* = 1.08	*w* = 1/[σ^2^(*F*_o_^2^) + (0.0242*P*)^2^ + 2.8471*P*] where *P* = (*F*_o_^2^ + 2*F*_c_^2^)/3
6065 reflections	(Δ/σ)_max_ = 0.002
319 parameters	Δρ_max_ = 0.62 e Å^−^^3^
0 restraints	Δρ_min_ = −0.61 e Å^−^^3^

### Special details

**Table:**

Experimental. Empirical absorption correction using spherical harmonics, implemented in SCALE3 ABSPACK scaling algorithm. CrysAlisPro (Agilent Technologies, 2012)
Geometry. All s.u.'s (except the s.u. in the dihedral angle between two l.s. planes) are estimated using the full covariance matrix. The cell s.u.'s are taken into account individually in the estimation of s.u.'s in distances, angles and torsion angles; correlations between s.u.'s in cell parameters are only used when they are defined by crystal symmetry. An approximate (isotropic) treatment of cell s.u.'s is used for estimating s.u.'s involving l.s. planes.
Refinement. Refinement of *F*^2^ against ALL reflections. The weighted *R*-factor *wR* and goodness of fit *S* are based on *F*^2^, conventional *R*-factors *R* are based on *F*, with *F* set to zero for negative *F*^2^. The threshold expression of *F*^2^ > σ(*F*^2^) is used only for calculating *R*-factors(gt) *etc*. and is not relevant to the choice of reflections for refinement. *R*-factors based on *F*^2^ are statistically about twice as large as those based on *F*, and *R*- factors based on ALL data will be even larger.

### Fractional atomic coordinates and isotropic or equivalent isotropic displacement parameters (Å^2^)

**Table:**

	*x*	*y*	*z*	*U*_iso_*/*U*_eq_	
Fe1	0.170301 (18)	0.73893 (3)	0.16941 (2)	0.01825 (7)	
P1	0.13712 (3)	0.49498 (4)	0.25844 (4)	0.01734 (11)	
S1	0.13878 (4)	0.39785 (5)	0.15519 (4)	0.02843 (13)	
N1	0.38708 (12)	0.51750 (17)	0.26266 (14)	0.0266 (4)	
C1	0.19359 (13)	0.62039 (17)	0.26970 (14)	0.0173 (4)	
C2	0.26568 (13)	0.64348 (18)	0.24522 (15)	0.0196 (4)	
C3	0.28607 (14)	0.75593 (19)	0.26702 (16)	0.0242 (5)	
H3	0.3307	0.7935	0.2581	0.029*	
C4	0.22953 (15)	0.80223 (19)	0.30379 (16)	0.0253 (5)	
H4	0.2297	0.8762	0.3241	0.030*	
C5	0.17231 (14)	0.72073 (17)	0.30565 (15)	0.0211 (4)	
H5	0.1274	0.7307	0.3270	0.025*	
C6	0.06407 (17)	0.7065 (2)	0.05634 (18)	0.0407 (7)	
H6	0.0244	0.6519	0.0537	0.049*	
C7	0.13415 (18)	0.6930 (2)	0.03003 (17)	0.0367 (6)	
H7	0.1500	0.6273	0.0070	0.044*	
C8	0.17626 (15)	0.7942 (2)	0.04407 (16)	0.0292 (5)	
H8	0.2253	0.8089	0.0319	0.035*	
C9	0.13258 (15)	0.8694 (2)	0.07938 (17)	0.0309 (5)	
H9	0.1471	0.9439	0.0954	0.037*	
C10	0.06381 (16)	0.8155 (3)	0.08696 (18)	0.0377 (6)	
H10	0.0239	0.8473	0.1090	0.045*	
C21	0.30886 (13)	0.57269 (19)	0.19659 (15)	0.0231 (5)	
H21A	0.3230	0.6180	0.1496	0.028*	
H21B	0.2704	0.5149	0.1605	0.028*	
C23	0.42053 (19)	0.4499 (3)	0.2013 (2)	0.0448 (7)	
H23A	0.4707	0.4127	0.2417	0.067*	
H23B	0.4331	0.4975	0.1554	0.067*	
H23C	0.3797	0.3950	0.1667	0.067*	
C24	0.36912 (17)	0.4455 (2)	0.3336 (2)	0.0409 (7)	
H24A	0.3253	0.3939	0.2999	0.061*	
H24B	0.3516	0.4907	0.3772	0.061*	
H24C	0.4187	0.4045	0.3703	0.061*	
C25	0.45051 (16)	0.6000 (2)	0.3157 (2)	0.0396 (6)	
H25A	0.4302	0.6429	0.3581	0.059*	
H25B	0.4618	0.6491	0.2699	0.059*	
H25C	0.5011	0.5619	0.3537	0.059*	
C111	0.17654 (13)	0.43111 (17)	0.37555 (15)	0.0186 (4)	
C112	0.23704 (14)	0.47914 (18)	0.45243 (15)	0.0214 (4)	
H112	0.2616	0.5462	0.4442	0.026*	
C113	0.26174 (15)	0.4291 (2)	0.54143 (16)	0.0269 (5)	
H113	0.3040	0.4611	0.5940	0.032*	
C114	0.22482 (15)	0.3329 (2)	0.55350 (16)	0.0289 (5)	
H114	0.2411	0.2995	0.6148	0.035*	
C115	0.16466 (15)	0.2848 (2)	0.47744 (17)	0.0285 (5)	
H115	0.1396	0.2185	0.4864	0.034*	
C116	0.14071 (14)	0.33311 (19)	0.38783 (16)	0.0240 (5)	
H116	0.0999	0.2993	0.3350	0.029*	
C121	0.03365 (13)	0.53257 (17)	0.24974 (15)	0.0204 (4)	
C122	0.01987 (14)	0.57870 (19)	0.32821 (16)	0.0243 (5)	
H122	0.0646	0.5903	0.3863	0.029*	
C123	−0.05875 (15)	0.6075 (2)	0.32155 (19)	0.0313 (5)	
H123	−0.0678	0.6403	0.3747	0.038*	
C124	−0.12427 (16)	0.5887 (2)	0.2378 (2)	0.0359 (6)	
H124	−0.1782	0.6088	0.2333	0.043*	
C125	−0.11120 (15)	0.5408 (2)	0.16082 (19)	0.0348 (6)	
H125	−0.1564	0.5272	0.1036	0.042*	
C126	−0.03235 (15)	0.51233 (19)	0.16629 (17)	0.0270 (5)	
H126	−0.0237	0.4791	0.1131	0.032*	
C100	0.40211 (16)	0.1117 (2)	0.40949 (18)	0.0339 (6)	
H100	0.4611	0.1343	0.4362	0.041*	
Cl1	0.39374 (5)	−0.02380 (6)	0.44430 (6)	0.04887 (18)	
Cl2	0.34498 (6)	0.19884 (7)	0.45463 (7)	0.0642 (3)	
Cl3	0.36757 (5)	0.12290 (10)	0.28457 (5)	0.0685 (3)	
I1	0.586539 (10)	0.293348 (13)	0.433624 (12)	0.03129 (6)	

### Atomic displacement parameters (Å^2^)

**Table:**

	*U*^11^	*U*^22^	*U*^33^	*U*^12^	*U*^13^	*U*^23^
Fe1	0.01707 (16)	0.01936 (16)	0.01709 (15)	−0.00029 (12)	0.00449 (12)	0.00296 (12)
P1	0.0202 (3)	0.0167 (3)	0.0162 (2)	−0.0016 (2)	0.0079 (2)	−0.0016 (2)
S1	0.0382 (3)	0.0255 (3)	0.0257 (3)	−0.0051 (3)	0.0164 (3)	−0.0105 (2)
N1	0.0222 (10)	0.0349 (11)	0.0263 (10)	0.0080 (9)	0.0128 (8)	0.0060 (9)
C1	0.0195 (10)	0.0176 (10)	0.0141 (9)	−0.0006 (8)	0.0050 (8)	0.0013 (8)
C2	0.0179 (10)	0.0214 (11)	0.0177 (10)	−0.0007 (9)	0.0041 (8)	0.0040 (8)
C3	0.0210 (11)	0.0237 (11)	0.0241 (11)	−0.0042 (9)	0.0031 (9)	0.0031 (9)
C4	0.0280 (12)	0.0191 (11)	0.0242 (11)	−0.0026 (9)	0.0037 (10)	−0.0022 (9)
C5	0.0246 (11)	0.0212 (11)	0.0167 (10)	0.0006 (9)	0.0064 (9)	−0.0001 (8)
C6	0.0298 (14)	0.0541 (18)	0.0255 (13)	−0.0149 (13)	−0.0061 (11)	0.0118 (12)
C7	0.0471 (16)	0.0378 (15)	0.0173 (11)	0.0080 (12)	0.0015 (11)	0.0004 (10)
C8	0.0251 (12)	0.0424 (15)	0.0198 (11)	0.0069 (11)	0.0077 (10)	0.0125 (10)
C9	0.0330 (13)	0.0300 (13)	0.0273 (12)	0.0069 (11)	0.0079 (10)	0.0132 (10)
C10	0.0225 (13)	0.0568 (18)	0.0320 (13)	0.0130 (12)	0.0073 (11)	0.0167 (12)
C21	0.0201 (11)	0.0285 (12)	0.0207 (10)	0.0026 (9)	0.0075 (9)	0.0040 (9)
C23	0.0430 (16)	0.0575 (19)	0.0407 (15)	0.0219 (14)	0.0232 (13)	−0.0007 (14)
C24	0.0346 (15)	0.0467 (16)	0.0480 (16)	0.0167 (13)	0.0229 (13)	0.0256 (13)
C25	0.0232 (13)	0.0520 (17)	0.0382 (14)	0.0023 (12)	0.0039 (11)	0.0032 (13)
C111	0.0208 (11)	0.0183 (10)	0.0200 (10)	0.0021 (8)	0.0113 (9)	0.0017 (8)
C112	0.0246 (12)	0.0196 (11)	0.0228 (10)	−0.0008 (9)	0.0117 (9)	−0.0018 (9)
C113	0.0295 (13)	0.0291 (12)	0.0204 (11)	0.0002 (10)	0.0066 (10)	−0.0013 (9)
C114	0.0347 (14)	0.0311 (13)	0.0225 (11)	0.0050 (11)	0.0121 (10)	0.0076 (10)
C115	0.0314 (13)	0.0252 (12)	0.0326 (13)	−0.0023 (10)	0.0158 (11)	0.0065 (10)
C116	0.0230 (12)	0.0228 (11)	0.0263 (11)	−0.0027 (9)	0.0087 (9)	0.0010 (9)
C121	0.0207 (11)	0.0182 (10)	0.0231 (10)	−0.0025 (9)	0.0087 (9)	0.0006 (8)
C122	0.0241 (12)	0.0250 (12)	0.0246 (11)	−0.0012 (9)	0.0096 (9)	0.0002 (9)
C123	0.0314 (13)	0.0277 (13)	0.0399 (14)	0.0026 (10)	0.0189 (12)	0.0000 (11)
C124	0.0249 (13)	0.0313 (14)	0.0527 (16)	0.0046 (11)	0.0150 (12)	0.0068 (12)
C125	0.0236 (13)	0.0340 (14)	0.0396 (14)	−0.0050 (11)	0.0020 (11)	0.0021 (11)
C126	0.0264 (12)	0.0258 (12)	0.0264 (12)	−0.0066 (10)	0.0064 (10)	−0.0026 (9)
C100	0.0264 (13)	0.0414 (15)	0.0296 (13)	0.0052 (11)	0.0044 (11)	0.0017 (11)
Cl1	0.0362 (4)	0.0370 (4)	0.0614 (5)	−0.0056 (3)	0.0022 (3)	−0.0025 (3)
Cl2	0.0818 (6)	0.0558 (5)	0.0758 (6)	0.0302 (5)	0.0536 (5)	0.0199 (4)
Cl3	0.0528 (5)	0.1143 (8)	0.0300 (4)	−0.0006 (5)	0.0042 (3)	0.0028 (4)
I1	0.02343 (9)	0.03225 (10)	0.03755 (10)	0.00095 (6)	0.00995 (7)	−0.00385 (7)

### Geometric parameters (Å, º)

**Table:**

Fe1—C2	2.017 (2)	C21—H21A	0.9900
Fe1—C1	2.017 (2)	C21—H21B	0.9900
Fe1—C8	2.027 (2)	C23—H23A	0.9800
Fe1—C7	2.030 (2)	C23—H23B	0.9800
Fe1—C5	2.035 (2)	C23—H23C	0.9800
Fe1—C9	2.036 (2)	C24—H24A	0.9800
Fe1—C3	2.039 (2)	C24—H24B	0.9800
Fe1—C10	2.053 (3)	C24—H24C	0.9800
Fe1—C6	2.056 (3)	C25—H25A	0.9800
Fe1—C4	2.056 (2)	C25—H25B	0.9800
P1—C1	1.792 (2)	C25—H25C	0.9800
P1—C111	1.814 (2)	C111—C112	1.385 (3)
P1—C121	1.818 (2)	C111—C116	1.389 (3)
P1—S1	1.9524 (7)	C112—C113	1.386 (3)
N1—C24	1.492 (3)	C112—H112	0.9500
N1—C23	1.495 (3)	C113—C114	1.380 (3)
N1—C25	1.497 (3)	C113—H113	0.9500
N1—C21	1.527 (3)	C114—C115	1.375 (4)
C1—C5	1.435 (3)	C114—H114	0.9500
C1—C2	1.453 (3)	C115—C116	1.385 (3)
C2—C3	1.424 (3)	C115—H115	0.9500
C2—C21	1.490 (3)	C116—H116	0.9500
C3—C4	1.403 (3)	C121—C126	1.388 (3)
C3—H3	0.9500	C121—C122	1.395 (3)
C4—C5	1.414 (3)	C122—C123	1.382 (3)
C4—H4	0.9500	C122—H122	0.9500
C5—H5	0.9500	C123—C124	1.383 (4)
C6—C10	1.405 (4)	C123—H123	0.9500
C6—C7	1.417 (4)	C124—C125	1.377 (4)
C6—H6	0.9500	C124—H124	0.9500
C7—C8	1.412 (4)	C125—C126	1.390 (3)
C7—H7	0.9500	C125—H125	0.9500
C8—C9	1.406 (3)	C126—H126	0.9500
C8—H8	0.9500	C100—Cl2	1.745 (3)
C9—C10	1.404 (4)	C100—Cl1	1.752 (3)
C9—H9	0.9500	C100—Cl3	1.753 (3)
C10—H10	0.9500	C100—H100	1.0000
			
C2—Fe1—C1	42.23 (8)	Fe1—C6—H6	126.7
C2—Fe1—C8	114.24 (9)	C8—C7—C6	108.0 (2)
C1—Fe1—C8	149.55 (9)	C8—C7—Fe1	69.50 (14)
C2—Fe1—C7	108.25 (10)	C6—C7—Fe1	70.69 (15)
C1—Fe1—C7	118.22 (10)	C8—C7—H7	126.0
C8—Fe1—C7	40.74 (11)	C6—C7—H7	126.0
C2—Fe1—C5	69.81 (9)	Fe1—C7—H7	125.4
C1—Fe1—C5	41.49 (8)	C9—C8—C7	107.7 (2)
C8—Fe1—C5	166.33 (10)	C9—C8—Fe1	70.09 (13)
C7—Fe1—C5	152.30 (10)	C7—C8—Fe1	69.76 (13)
C2—Fe1—C9	146.24 (9)	C9—C8—H8	126.2
C1—Fe1—C9	169.62 (9)	C7—C8—H8	126.2
C8—Fe1—C9	40.51 (10)	Fe1—C8—H8	125.6
C7—Fe1—C9	68.06 (11)	C10—C9—C8	108.3 (2)
C5—Fe1—C9	129.32 (10)	C10—C9—Fe1	70.58 (14)
C2—Fe1—C3	41.11 (9)	C8—C9—Fe1	69.41 (13)
C1—Fe1—C3	69.65 (9)	C10—C9—H9	125.8
C8—Fe1—C3	105.52 (10)	C8—C9—H9	125.8
C7—Fe1—C3	129.23 (11)	Fe1—C9—H9	125.8
C5—Fe1—C3	68.38 (9)	C9—C10—C6	108.3 (2)
C9—Fe1—C3	113.61 (10)	C9—C10—Fe1	69.25 (14)
C2—Fe1—C10	171.76 (11)	C6—C10—Fe1	70.12 (15)
C1—Fe1—C10	132.36 (10)	C9—C10—H10	125.8
C8—Fe1—C10	67.91 (10)	C6—C10—H10	125.8
C7—Fe1—C10	67.79 (11)	Fe1—C10—H10	126.4
C5—Fe1—C10	110.07 (10)	C2—C21—N1	115.32 (18)
C9—Fe1—C10	40.17 (10)	C2—C21—H21A	108.4
C3—Fe1—C10	147.00 (11)	N1—C21—H21A	108.4
C2—Fe1—C6	132.44 (11)	C2—C21—H21B	108.4
C1—Fe1—C6	111.10 (10)	N1—C21—H21B	108.4
C8—Fe1—C6	68.21 (10)	H21A—C21—H21B	107.5
C7—Fe1—C6	40.57 (12)	N1—C23—H23A	109.5
C5—Fe1—C6	119.62 (10)	N1—C23—H23B	109.5
C9—Fe1—C6	67.65 (11)	H23A—C23—H23B	109.5
C3—Fe1—C6	169.41 (11)	N1—C23—H23C	109.5
C10—Fe1—C6	39.98 (12)	H23A—C23—H23C	109.5
C2—Fe1—C4	68.76 (9)	H23B—C23—H23C	109.5
C1—Fe1—C4	69.09 (9)	N1—C24—H24A	109.5
C8—Fe1—C4	127.30 (10)	N1—C24—H24B	109.5
C7—Fe1—C4	166.46 (11)	H24A—C24—H24B	109.5
C5—Fe1—C4	40.42 (9)	N1—C24—H24C	109.5
C9—Fe1—C4	106.63 (10)	H24A—C24—H24C	109.5
C3—Fe1—C4	40.07 (9)	H24B—C24—H24C	109.5
C10—Fe1—C4	116.83 (11)	N1—C25—H25A	109.5
C6—Fe1—C4	150.50 (11)	N1—C25—H25B	109.5
C1—P1—C111	105.46 (10)	H25A—C25—H25B	109.5
C1—P1—C121	106.77 (10)	N1—C25—H25C	109.5
C111—P1—C121	101.86 (9)	H25A—C25—H25C	109.5
C1—P1—S1	115.51 (7)	H25B—C25—H25C	109.5
C111—P1—S1	113.33 (7)	C112—C111—C116	120.0 (2)
C121—P1—S1	112.72 (8)	C112—C111—P1	122.49 (16)
C24—N1—C23	109.6 (2)	C116—C111—P1	117.47 (17)
C24—N1—C25	108.6 (2)	C111—C112—C113	119.9 (2)
C23—N1—C25	108.7 (2)	C111—C112—H112	120.1
C24—N1—C21	110.85 (18)	C113—C112—H112	120.1
C23—N1—C21	107.32 (19)	C114—C113—C112	119.9 (2)
C25—N1—C21	111.69 (19)	C114—C113—H113	120.1
C5—C1—C2	106.79 (18)	C112—C113—H113	120.1
C5—C1—P1	123.78 (16)	C115—C114—C113	120.5 (2)
C2—C1—P1	129.43 (16)	C115—C114—H114	119.7
C5—C1—Fe1	69.95 (12)	C113—C114—H114	119.7
C2—C1—Fe1	68.88 (11)	C114—C115—C116	120.0 (2)
P1—C1—Fe1	125.51 (11)	C114—C115—H115	120.0
C3—C2—C1	107.25 (19)	C116—C115—H115	120.0
C3—C2—C21	122.72 (19)	C115—C116—C111	119.8 (2)
C1—C2—C21	129.7 (2)	C115—C116—H116	120.1
C3—C2—Fe1	70.31 (13)	C111—C116—H116	120.1
C1—C2—Fe1	68.89 (12)	C126—C121—C122	119.6 (2)
C21—C2—Fe1	121.12 (15)	C126—C121—P1	120.34 (17)
C4—C3—C2	108.9 (2)	C122—C121—P1	120.05 (17)
C4—C3—Fe1	70.62 (13)	C123—C122—C121	120.1 (2)
C2—C3—Fe1	68.59 (12)	C123—C122—H122	120.0
C4—C3—H3	125.6	C121—C122—H122	120.0
C2—C3—H3	125.6	C122—C123—C124	120.2 (2)
Fe1—C3—H3	126.8	C122—C123—H123	119.9
C3—C4—C5	108.8 (2)	C124—C123—H123	119.9
C3—C4—Fe1	69.31 (13)	C125—C124—C123	119.9 (2)
C5—C4—Fe1	68.99 (12)	C125—C124—H124	120.0
C3—C4—H4	125.6	C123—C124—H124	120.0
C5—C4—H4	125.6	C124—C125—C126	120.5 (2)
Fe1—C4—H4	127.7	C124—C125—H125	119.8
C4—C5—C1	108.33 (19)	C126—C125—H125	119.8
C4—C5—Fe1	70.59 (12)	C121—C126—C125	119.7 (2)
C1—C5—Fe1	68.56 (11)	C121—C126—H126	120.1
C4—C5—H5	125.8	C125—C126—H126	120.1
C1—C5—H5	125.8	Cl2—C100—Cl1	109.90 (14)
Fe1—C5—H5	126.6	Cl2—C100—Cl3	109.62 (14)
C10—C6—C7	107.6 (2)	Cl1—C100—Cl3	110.86 (15)
C10—C6—Fe1	69.89 (15)	Cl2—C100—H100	108.8
C7—C6—Fe1	68.74 (15)	Cl1—C100—H100	108.8
C10—C6—H6	126.2	Cl3—C100—H100	108.8
C7—C6—H6	126.2		

### Hydrogen-bond geometry (Å, º)

**Table:**

*D*—H···*A*	*D*—H	H···*A*	*D*···*A*	*D*—H···*A*
C24—H24*C*···I1	0.98	3.05	4.001 (3)	163
C100—H100···I1	1.00	2.93	3.810 (3)	147

### Comparison of the Cp—C—NMe_3_ framework (Å, °) in the title compound and related structures in the CSD.

N1···Cp is the distance of the N atom from the Cp ring, C21···Cp is the distance of the C21 atom from the Cp ring and Ang1 is the dihedral angle between the Cp ring and the plane defined by C2—C21—N1.

**Table:**

Reference	C2—C21	C21—N1	N1···Cp	C21···Cp	Ang
This study	1.490 (3)	1.527 (3)	1.182 (2)	-0.128 (2)	83.2 (2)
ASAZIE	1.476	1.530	1.253	-0.096	87.49
BUBCOQ	1.505	1.520	1.256	-0.106	90.0
BUBCUW(1)	1.518	1.531	1.260	-0.095	84.38
BUBCUW(2)	1.494	1.525	1.311	-0.048	87.72
DEHHUU	1.482	1.524	1.220	-0.111	90.0
EDUQUP	1.493	1.525	1.294	-0.072	88.68
HABDUL(1)	1.493	1.520	1.167	-0.147	87.20
HABDUL(2)	1.467	1.472	1.270	-0.033	85.45
HABFAT(1)	1.460	1.530	1.233	-0.125	86.58
HABFAT(2)	1.478	1.540	1.125	-0.167	84.17
HABFAT(3)	1.499	1.526	1.309	-0.063	88.73
HIZFOM(1)	1.471	1.519	1.327	-0.029	81.08
HIZFOM(2)	1.447	1.525	1.425	-0.032	90.0
HIZFOM(3)	1.432	1.515	1.393	-0.042	83.97
HIZFOM(4)	1.336	1.529	1.335	-0.007	90.0
IBIROB(1)	1.493	1.529	1.197	-0.142	88.83
IBIROB(2)	1.470	1.537	1.324	-0.060	82.55
IGEPUG(1)	1.519	1.520	1.248	-0.066	77.69
IGEPUG(2)	1.522	1.523	0.999	-0.274	85.92
IGEPUG(3)	1.514	1.533	1.914	-0.426	81.02
IGEPUG(4)	1.516	1.533	1.152	-0.163	83.23
IGEQAN(1)	1.462	1.533	1.251	-0.106	82.66
IGEQAN(2)	1.481	1.543	1.216	-0.123	87.22
IKONOL	1.485	1.522	1.223	-0.100	84.09
IKONOL01	1.484	1.514	1.223	-0.097	90.0
IKONUR	1.495	1.526	1.177	-0.134	86.93
IKUZOD(1)	1.493	1.530	1.272	-0.073	80.32
IKUZOD(2)	1.487	1.528	1.316	-0.050	82.74
IKUZUJ(1)	1.484	1.535	1.290	-0.072	88.63
IKUZUJ(2)	1.489	1.537	1.256	-0.094	87.41
IQUCIG	1.485	1.528	1.263	-0.083	79.62
JUHXEP	1.471	1.516	1.283	-0.066	88.29
JUJDOH	1.482	1.536	1.330	-0.056	88.54
JUJDOH01	1.488	1.510	1.327	-0.033	87.68
JUJFEZ	1.485	1.530	1.225	-0.111	88.50
LEJHIR	1.488	1.521	1.129	-0.141	76.82
LEJHOX(1)	1.502	1.523	1.200	-0.120	83.12
LEJHOX(2)	1.484	1.526	1.203	-0.127	86.84
LIFWUS(1)	1.509	1.536	1.127	-0.166	78.64
LIFWUS(2)	1.485	1.544	1.101	-0.127	69.81
LIFXAZ	1.494	1.525	1.125	-0.129	70.44
NAGHOU	1.501	1.527	1.256	-0.060	77.63
NAGHUA(1)	1.489	1.526	1.235	-0.097	79.70
NAGHUA(2)	1.495	1.528	1.182	-0.129	79.44
NATZEO	1.459	1.549	1.295	-0.074	89.24
NEYSIT	1.486	1.525	1.256	-0.090	86.76
SAZWIA	1.472	1.530	1.188	-0.119	80.76
WASGED(1)	1.489	1.533	1.181	-0.150	88.52
WASGED(2)	1.488	1.523	1.239	-0.101	88.53
WASGED(3)	1.479	1.518	1.241	-0.093	88.22
XAJNIF(1)	1.481	1.519	1.318	-0.054	87.91
XAJNIF(2)	1.488	1.532	1.325	-0.049	87.05
XEQKIN	1.497	1.531	1.378	-0.012	84.17
YOVGOF	1.488	1.524	1.265	-0.057	75.89

Notes: ASAZIE (Bai *et al.*, 2011); BUBCOQ (Zhuji *et al.*, 1982); BUBCUW (Yongmao *et al.*, 1982); DEHHUU (Volkov *et al.*, 2006); EDUQUP (Reynes *et al.*, 2002); HABDUL (Xu *et al.*, 2010); HABFAT (Xu *et al.*, 2010) ; HIZFOM (Selvapalam *et al.*, 2007); IBIROB (Hu *et al.*, 2004); IGEPUG (Li *et al.*, 2009); IGEQAN (Li *et al.*, 2009); IKONOL (Ballester *et al.*, 2003); IKOOL01 (Herbstein & Kapon, 2008); IKONUR (Ballester *et al.*, 2003); IKUZOD (Volkov *et al.*, 2003); IKUZUJ (Volkov *et al.*, 2003); IQUCIG (Blake *et al.*, 2004); JUHXEP (Pullen *et al.*, 1998); JUJDOH (Pullen *et al.*, 1998); JUJDOH01 (Pullen *et al.*, 1998); JUJFEZ (Pullen *et al.*, 1998); LEJHIR (Ferguson *et al.*, 1994); LEJHOX (Ferguson *et al.*, 1994); LIFWUS (Malezieux *et al.*, 1994); LIFXAZ (Malezieux *et al.*, 1994); NAGHOU (Broomsgrove *et al.*, 2010); NAGHUA (Broomsgrove *et al.*, 2010) ; NATZEO (Hong *et al.*, 2005); NEYSIT (Chohan *et al.*, 1997); SAZWIA (Sharma *et al.*, 2006); WASGED (Volkov *et al.*, 2005); XAJNIF (Hosmane *et al.*, 1998); XEQKIN (Deck *et al.*, 2000); YOVGOF (Veya & Kochi, 1995).
